# Clinical predictors of drug-resistant tuberculosis in Mexico

**DOI:** 10.1371/journal.pone.0220946

**Published:** 2019-08-15

**Authors:** Samantha Flores-Treviño, Eduardo Rodríguez-Noriega, Elvira Garza-González, Esteban González-Díaz, Sergio Esparza-Ahumada, Rodrigo Escobedo-Sánchez, Héctor R. Pérez-Gómez, Gerardo León-Garnica, Rayo Morfín-Otero

**Affiliations:** 1 Servicio de Gastroenterología, Hospital Universitario Dr. José Eleuterio González, Universidad Autónoma de Nuevo León, Monterrey, Nuevo León, México; 2 Hospital Civil de Guadalajara, Fray Antonio Alcalde, Instituto de Patología Infecciosa y Experimental, Centro Universitario de Ciencias de la Salud, Universidad de Guadalajara, Guadalajara, Jalisco, México; Jamia Hamdard, INDIA

## Abstract

Drug-resistant tuberculosis (DR-TB) remains a major global health problem. Early treatment of TB is critical; in the absence of rapid- susceptibility testing, the empiric selection of drugs should be guided by clinical data. This study aimed to determine the clinical predictors of DR-TB. From September 2010 to August 2017, sociodemographic and clinical characteristics were collected from 144 patients with tuberculosis at the Hospital Civil de Guadalajara, Mexico. Isolates were subjected to drug-susceptibility testing. Clinical predictors of DR-TB were determined using univariate and multivariate analysis. Any drug, isoniazid, and rifampin resistance rates were 47.7, 23.0, and 11.6%, respectively. The visualization of cavities and nodules through either chest radiography or computed tomography were independent predictors of DR-TB. In conclusion, early detection of DR-TB in this population could be based on multiple cavities being observed using chest imaging. This study’s results can be applied to future patients with TB in our community to optimize the DR-TB diagnostic process.

## Introduction

Tuberculosis (TB) continues to be a severe global health problem, which is compounded by the appearance of drug resistance. TB is currently the ninth leading cause of death worldwide and the leading cause from an infectious disease, ranking above HIV/AIDS. Furthermore, it is the main cause of death related to antimicrobial resistance and HIV infection. A review by the World Health Organization (WHO) Global TB Programme estimated that there were 10.4 million new cases of TB in 2016, including 600,000 new cases with rifampicin (RIF) resistance, as well as 1.7 million deaths [1]. In the same year in Mexico, there were 16,913 new cases of pulmonary TB, and the 2015 mortality rate per 100,000 of the population was 1.7. In the western state of Jalisco, the TB mortality rate was 1.6 per 100,000 [2]. These data indicate that TB is a major global health problem that deserves special attention. Multidrug-resistant TB (MDR-TB), resistant to isoniazid (INH) and RIF, complicates treatment, posing a challenge for clinicians. Currently, it is a serious public health issue, especially in developing countries [3].

The risk of exposure and progression to active TB is affected by several risk factors, such as HIV, smoking, diabetes mellitus (DM), alcohol use, malnourishment, immunosuppressive drug usage, young age, overcrowding, housing conditions, and economic deprivation [4]. After 6 months of first-line therapy for drug-susceptible TB, 5% of patients will relapse; moreover, the relapse rate will be 20% after 4 months of short-course therapy. In the search for risk factors associated with relapses, part of a relapse model for TB was formed by the presence of a higher minimum inhibitory concentration for INH or RIF during pretreatment, *Mycobacterium tuberculosis* isolates in combination with a positive culture at 8 weeks, underweight, white race, cavitation detected in a chest radiograph, and bilateral disease [5].

In Mexican patients, studies on predictors for TB infection have indicated mostly socioeconomic factors, such as poor nutrition, TB family history or living with a relative with TB, history of incarceration, poorly controlled DM, foreign birth, lower socioeconomic status, and marital status [6–9]. Moreover, being male, aged 20 to 59 years, having had contact with a person with TB, history of previous TB, prior TB treatment, and HIV infection have been associated with DR-TB infections [10–13]. Nevertheless, determining clinical predictors of TB infection would greatly aid current treatments, especially in our settings. The initial treatment of TB is critical; in the absence of rapid-susceptibility testing, the empiric selection of drugs should be guided by clinical data. Therefore, this study aimed to identify potential clinical predictors of DR-TB in patients obtained over a 7-year period from Guadalajara, Jalisco to more effectively approach the diagnosis of either DR-TB or MDR-TB.

## Materials and methods

### Ethics statement

The local ethics committee (Comité de Ética en Investigación, Hospital Civil de Guadalajara Fray Antonio Alcalde, Universidad de Guadalajara, Jalisco, Mexico) approved the study (123/17), and each patient provided written informed consent to participate.

### Site

The Hospital Civil de Guadalajara Fray Antonio Alcalde is an 899-bed tertiary-care teaching hospital located in Guadalajara, the second largest city in western Mexico. The hospital provides care to adult and pediatric patients in 31 wards situated in three connected buildings. It serves a population spanning the greater Guadalajara metropolitan area, including its seven municipalities (approximately 4.0 million habitants) and surroundings states. From 2010 to 2017, there were 240,000 discharges and a daily occupancy rate of 94.7%, with patients having a mean length of stay of 7.2 days. Over 1,000 patients with TB were admitted between 2010 and 2017, mostly from Jalisco and the surrounding western states.

### Data collection

The collected sociodemographic information included age, sex, place of residence, employment status, education level (none, primary [first to sixth grade], secondary, or tertiary [preparatory or college/university]), and marital status (single or married).

Clinical characteristics included the type of sample (pulmonary or extrapulmonary), site of TB (pulmonary or extrapulmonary), treatment category (new or retreatment), HIV infection, history of previous TB, history of previous TB exposure, and presenting symptoms (fever, weight loss, cough, chills, hemoptysis, expectoration, lymphadenopathy, diaphoresis, dyspnea, dysphonia, dysphagia, diarrhea, headache, seizures, altered levels of consciousness, back pain, abdominal pain, chest pain, cachexia, poor general condition [including the presence of asthenia, adynamia, hyporexia, and anorexia]), and time in weeks between the appearance of first symptoms and receiving attention.

Radiological characteristics included chest radiography patterns (cavities, miliary, pleural effusion, consolidation, nodule, or interstitial) and computed tomography (CT) characteristics (apical asymmetry, cavities, miliary, or nodule).

Histories of potential co-morbidities were also included, such as alcohol and drug use, smoking, history of incarceration, and DM.

### Clinical specimens and isolates

From September 2010 to August 2017, specimens (*N* = 1,175) were referred to the collaborative mycobacteriology laboratory for further testing.

All samples were decontaminated using the Petroff method and cultured on Löwestein–Jensen slants at 37°C. Smears stained using the Ziehl–Neelsen method were examined for the presence of acid-fast bacilli. Isolates were identified by conventional biochemical methods including niacin production and nitrate reduction tests. One isolate per patient was assayed. Mycobacteria were first subjected to enzymatic lysis with lysozyme and proteinase K. Using phenol extraction and ethanol precipitation, DNA was then obtained. Species identification was performed using multiplex PCR amplification of the *cfp32* (Rv0577), RD9 (Rv2073c) and RD12 (Rv3120) genes [14].

### Drug-susceptibility testing

Drug-susceptibility testing of the *M*. *tuberculosis* isolates was performed as previously described, using the indirect proportion method on Löwestein-Jensen slants with the following critical concentrations: streptomycin (STR) 4.0 μg/mL, INH 0.2 μg/mL, RIF 40 μg/mL, and ethambutol (EMB) 2.0 μg/mL. Strain H37Ra was used as a control [14].

### Statistical analysis

Descriptive statistics were used to summarize patients’ sociodemographic, clinical, radiological, and mycobacteriological characteristics. Univariate analyses, including chi-square analysis, were used to identify risk factors associated with MDR-TB. After considering variables shown by univariate analysis to be suggestively associated, multivariate logistic regression was performed to assess the independent predictors of each MDR-TB category. Odds ratios (ORs) and 95% confidence intervals (CIs) were estimated. The statistical analyses were conducted using SPSS version 25.0 (IBM, SPSS Inc., Chicago, IL, USA). A *p*-value < .05 was considered statistically significant.

## Results

### Patients and strains

During the 7-year period, 1,175 clinical samples were initially processed, of which 171 (14.6%) patients had a positive mycobacterial culture; 144 (81.4%) isolates were identified as *M*. *tuberculosis* through biochemical and molecular tests. Sputum smear microscopy was positive in all 144 (100.0%) samples.

### Clinical characteristics

The sample types were mostly pulmonary (*n* = 138, 95.8%). A total of 138 (95.8%) patients had pulmonary TB; the majority were new cases (*n* = 104, 72.2%). Fifteen (10.4%) patients were HIV-infected, 40 (27.8%) had histories of previous TB, and 46 (31.9%) had histories of previous TB exposure.

The frequency of symptoms presented in patients included poor general condition (*n* = 123, 85.4%), cough (*n =* 120, 83.3%), weight loss (*n =* 115, 79.9%), fever (*n =* 113, 78.5%), expectoration (*n =* 110, 76.4%), chills (*n =* 105, 72.9%), diaphoresis (*n =* 95, 66.0%), dyspnea (*n =* 92, 63.9%), hemoptysis (*n =* 40, 27.8%), chest pain (*n =* 32, 22.2%), diarrhea (*n =* 30, 20.8%), dysphonia (*n =* 20, 13.9%), abdominal pain (*n =* 18, 12.5%), dysphagia (*n =* 16, 11.1%), lymphadenopathy (*n =* 14, 9.7%), altered levels of consciousness (*n =* 12, 8.3%), back pain (*n =* 11, 7.6%), headache (*n =* 9, 6.3%), cachexia (*n =* 5, 3.5%), and seizures (*n =* 3, 2.1%). Time elapsed between the appearance of first symptoms and receiving attention was 23.7 ± 29.0 weeks.

Regarding radiological characteristics, chest radiography was available in 132 (91.7%) patients, and the resulting patterns included nodule (*n =* 77, 53.5%), cavities (*n =* 63, 43.8%), interstitial (*n =* 54, 37.5%), consolidation (*n =* 41, 28.5%), pleural effusion (*n =* 15, 10.4%), and miliary (*n =* 14, 9.7%). CT was available in 129 (89.6%) patients and its characteristics included nodule (*n =* 99, 68.8%), apical asymmetry (*n =* 95, 66.0%), cavities (*n =* 84, 58.3%), and miliary (*n =* 16, 11.1%) patterns.

Co-morbidity frequencies were determined for smoking (*n =* 91, 63.2%), alcohol use (*n =* 77, 53.5%), drug use (*n =* 57, 39.6%), DM (*n =* 38, 26.4%), and history of incarceration (*n =* 24, 16.7%).

### Drug resistance

Drug-susceptibility testing was performed on 86 (62.3%) isolates. Drug resistance was detected in 41 (47.7%) patients, INH resistance was detected in 20 (23.0%) patients, and RIF and MDR resistance were each detected in 10 (11.6%) patients.

### Clinical predictors of DR-TB

Univariate analysis was performed on data with drug-susceptibility results, in which three categories were included: DR versus susceptible isolates, INH-resistant versus susceptible strains, and RIF-resistant versus susceptible isolates.

Infection with DR-TB strains was more frequent in patients who presented with fever, chest pain, diarrhea, back pain, headache, cachexia, and seizures as well as who exhibited cavities or nodules in chest radiography (OR = 2.88; 95% CI = 1.173–7.108; *p* = .019) or in chest CT tests (OR = 2.559; 95% CI = 1.071–6.114; *p* = .033) (S1 Table).

INH-resistant *M*. *tuberculosis* infection was more frequent in patients with histories of previous TB (OR = 3.097; 95% CI = 1.073–8.937; *p* = .032), fever, chest pain, or diarrhea (S1 Table). Furthermore, RIF-resistant *M*. *tuberculosis* infection was more frequent in patients with a history of previous TB, fever, chills, and nodules in chest radiographs or chest CT tests (S1 Table).

In the multivariate analysis, the presence of cavitation was more frequent in patients infected with DR isolates (OR = 2.559; 95% CI = 1.071–6.114; *p* = .034), whereas nodules and cavitation were more frequent in patients infected with INH-resistant isolates (OR = 5.250; 95% CI = 1.256–21.945; *p* = .023) and RIF-resistant isolates (OR = 6.086; 95% CI = 1.194–31.011; *p* = .030) (Table 1).

**Table 1:** Multivariate analysis of patients’ characteristics by DR, INH resistant, and MDR/RIF resistant categories.

Variable	OR	95% CI	*p* value
DR			
Cavitation in chest radiography	2.559	1.071–6.114	**0.034**
INH resistance			
Nodules/cavitation in chest radiography	5.250	1.256–21.945	**0.023**
MDR/RIF resistance			
Nodules/cavitation in chest radiography	6.086	1.194–31.011	**0.030**

CI: confidence intervals; DR: drug resistance; INH: isoniazid; MDR: multidrug resistant; RIF: rifampicin; OR: odds ratio. Numbers in bold indicate a statistically significant p-value (*p* < 0.05).

## Discussion

A retrospective study was performed on data from patients with TB collected from a 7-year period to identify potential clinical predictors of DR-TB in our population; this was to enable a more effective approach to the diagnosis of either DR-TB or MDR-TB. Our results indicated that no sociodemographic characteristics were detected as risk factors associated with DR-TB. However, factors such as male sex, being 20 to 59 years of age, and foreign birth have been associated with DR-TB previously [10]. Having any contact with a person with TB has been associated with INH resistance [11]. Nevertheless, that was not our case. Also, exposure to certain environmental conditions such as poor nutrition, family history of TB, foreign birth [6], and lower socioeconomic status [7] have been associated with TB infection. History of previous TB [10] and prior TB treatment [12, 13] have been associated with DR-TB infection. However, our results showed that a history of previous TB was associated with INH susceptibility and it also was an independent predictor of it. Living with a relative with TB, history of incarceration, and poorly controlled DM were risk factors for having latent TB infection in persons with DM [8]. Marital status has also reported being independently associated with TB status [9]. HIV infection was not associated with DR-TB, as has been reported before [10]. In addition, mortality was more frequent in patients who had symptoms of altered level of consciousness, back pain, and cachexia. The first two were also independent predictors of mortality. Indeed, cachexia has also been associated with mortality in TB patients [15].

Chest imaging could guide the diagnostic process by early detection of MDR-TB. In this study, we have found that the presence of cavitation and nodules in chest x-rays or chest CTs suggests the existence of at least one type of DR in the patient. This finding forms part of the relapse model developed by Colangeli et al., which predicts relapse after TB therapy [5]. When a pretreatment *M*. *tuberculosis* isolate has a higher minimal inhibitory concentration for INH and RIF, there is a higher risk of relapse. This data was combined with the presence of a positive culture at eight weeks, being underweight, cavitation on chest radiograph, bilateral disease, and white race. The score has a sensitivity of 70% and a specificity of 100% [5].

The interest in using radiological studies to anticipate MDR-TB has included the presence of nodules, reticulonodular densities, consolidation, cavities, masses, bronchiectasis, fibrosis, calcification, nodes, atelectasis, bullae, and emphysema [16–20]. In a review of evidence published through January 2018, the prevalence of cavity lesions in MDR-TB is 70%, with a mean cavity number of ≥3; no differences were found between new MDR-TB and previously treated MDR-TB [21]. Current literature suggests that thick-walled multiple cavity lesions present the most promising radiological sign for MDR-TB diagnosis [20, 21]. Certainly, in our patients, DR-TB infection was more frequent in those patients who showed a cavity pattern in either their chest radiography or CT test. Also, the visualization of cavities by either chest radiography or CT test was an independent predictor of DR-TB. Significant differences have been detected in the frequency of multiple cavities, nodules, and bronchial dilatation shown on CT scans in MDR-TB patients [16]. Thus, the observation of multiple cavities in our population should suggest that the patient is infected with DR-TB instead of a susceptible strain. A chest radiography pattern suggestive of TB disease was more likely to present in patients infected with drug-susceptible strains (either to INH or RIF), and it showed to be an independent predictor of drug-susceptible TB strains. Active TB lesions in the forms of infiltrate, nodules, and ground-glass opacity [16, 20] have reported being more frequent in patients infected with drug-susceptible *M*. *tuberculosis* strains. In addition, mortality was more frequent in patients who had a chest radiography nodule pattern and an apical asymmetry pattern on CT, which were also independent predictors of mortality.

Several co-morbidities have been determined as risk factors for TB infection, such as DM [6, 8, 9, 22], smoking [9], and drug use [12]. Patients with a prior diagnosis of DM had a greater likelihood of failing treatment [23]. Consequently, co-morbid malignancies have been associated with increased risk of in-hospital death among pulmonary TB patients [24]. However, in the present study, co-morbidities such as smoking, alcohol, and drug use, DM and history of incarceration were not associated with DR-TB infection, as has been reported previously [10, 12]. Indeed, their frequency was almost equal among patients infected with susceptible and DR-TB.

In Mexico, the evolution of DR-TB has been reported since 1995 [25]. From 1995 to 2013 (15 studies), the overall resistance fluctuated between 9.7% and 72.2%. INH resistance fluctuated between 3.8% and 86.7%, RIF resistance between 0.84% and 69.2%, and MDR between 2.1% and 66.7%, with drug associated-mutation rates (2001–2013) of 1.6–50.6% [25]. In 2015, in 105 *M*. *tuberculosis* isolates from our hospital (Jalisco), INH resistance was found to be 40%, RIF resistance was 20%, and MDR was 19% [26]. In 2017, also from the state of Jalisco, 63 isolates from 2012 to 2013 showed monoresistance of 26.9%, polyresistance of 14.2%, and MDR of 7.9% [27]. Presently, in Jalisco DR-TB and MDR rates were 47.7% and 11.6%, respectively, which are higher than those reported for Estado de Mexico (31% and 12%, respectively) (23) and Veracruz (17% and 6%, respectively) [28]. The mean DR and MDR rates reported for the country were 37.5% and 20.6%, respectively [25], which are slightly different from those reported in Jalisco.

Susceptible TB is cured with a six-month course of antibiotics divided into two periods: an initial two-month period in which four anti-tuberculosis drugs are given daily for six days a week, followed by a four-month phase in which two anti-tuberculosis drugs are given three days a week. In Mexico, there is free universal access to this regimen. MDR-TB requires an expensive and prolonged regimen with new drugs, something that most countries still do not have.

All our patients had previous treatment for TB (EMB/INH/PZA/RIF), and half of them were treated with levofloxacin. Use of later-generation fluoroquinolones, such as levofloxacin, moxifloxacin, or gatifloxacin has significantly reduced patient mortality and improved treatment outcomes for DR-TB patients in comparison to lack of any fluoroquinolone treatment or treatment with an earlier-generation fluoroquinolone, such as ciprofloxacin or ofloxacin [29]. However, our results showed that infection of DR-TB strains was more frequent in patients treated with levofloxacin. Treatment with moxifloxacin did not reflect these same results though barely 6.9% of our patients were treated with it. Levofloxacin treatment was an independent predictor of DR-TB though it was not associated with higher mortality. Treatment with quinolones, linezolid, and meropenem, were also associated with mortality. The latter two were also independent predictors of mortality.

In our study, only a small proportion of the culture-positive specimens was obtained from re-treatment cases, which can be explained because after diagnosis and management, patients are referred to the closest TB clinic which is run by the Mexican national TB program. Thus, our data do not reflect the relapse rates observed in the region.

This study has several limitations, including the selection of patients that need hospitalization, short-term follow-up, no end-of-treatment radiography, and the lack of investigations of infections in contacts.

In conclusion, DR-TB can be predicted early in our population through the observation of multiple cavities by chest imaging. In patients with new or previously treated TB, the presence of cavitation and nodules in chest imaging demands the consideration of using an initial regimen that will cover MDR-TB. Patients and relatives should be informed of the risk of transmission of close contacts [30]. Moreover, the visualization of a nodule pattern on chest radiography or an apical asymmetry pattern on CT should suggest higher mortality in the patient. Likewise, the presence of symptoms such as altered level of consciousness or back pain should also suggest higher mortality in TB patients. Our results could apply to future TB patients in our community to optimize the DR-TB diagnostic process.

## Supporting information

S1 Table Sociodemographic, clinical, and radiological characteristics of patients with DR-TB infections. Patients infected with DR-TB, INH-resistant or MDR/RIF-resistant were compared against susceptible isolates using univariate analysis.
(DOCX)
