# Supplementary material for: Clinical predictors of drug-resistant tuberculosis in Mexico
Source: PLoS One. 2019 Aug 15;14(8):e0220946. doi: 10.1371/journal.pone.0220946 (PMC6695153; doi:10.1371/journal.pone.0220946)
Supplement: S1 Table — Patients infected with DR-TB, INH-resistant or MDR/RIF-resistant were compared against susceptible isolates using univariate analysis. (DOCX) [file pone.0220946.s001.docx]

**S1 Table. Sociodemographic, clinical, and radiological characteristics of patients with DR-TB infections. Patients infected with DR-TB, INH-resistant or MDR/RIF-resistant were compared against susceptible isolates using univariate analysis.**

| **Characteristic** | **No. (% or range)**  ***N* = 144** | **DR vs. S**  **No. (% or range) *N* = 86** | | | | **INH-R vs. S**  **No. (% or range) *N* = 87** | | | | **MDR/RIF-R vs. S**  **No. (% or range) *N* = 86** | | | |
| --- | --- | --- | --- | --- | --- | --- | --- | --- | --- | --- | --- | --- | --- |
|  |  | **R (*n =* 41)** | **S (*n =* 45)** | **OR**  **(95% CI)** | ***p*** | **R (*n =* 20)** | **S (*n =* 67)** | **OR**  **(95% CI)** | ***p*** | **R (*n =* 10)** | **S (*n =* 76)** | **OR**  **(95% CI)** | ***p*** |
| Sociodemographic data | | | | | | | | | | | | | |
| Age in years ± SD | 44.1 ± 17.4 | 43.9 ± 16.0 | 43.6 ± 17.7 |  | 0.617 | 43.8 ± 13.8 | 43.6 ± 17.7 |  | 0.865 | 47.3 ± 14.4 | 43.1 ± 17.2 |  | 0.788 |
| Male | 112 (77.8) | 34 (82.9) | 34 (75.6) | 0.636 (0.220–1.837) | 0.401 | 17 (85.0) | 51 (85.1) | 0.563 (0.146–2.169) | 0.399 | 10 (100.0) | 57 (75.0) | 0.851 (0.770–0.940) | 0.073 |
| Clinical characteristics | | | | | | | | | | | | | |
| Pulmonary | 138 (95.8) | 39 (95.1) | 43 (45.6) |  |  | 19 (95.0) | 64 (95.5) |  |  | 9 (90.0) | 73 (96.1) |  |  |
| Extrapulmonary | 6 (4.2) | 2 (4.9) | 2 (4.4) |  |  | 1 (5.0) | 3 (4.5) |  |  | 1 (10.0) | 3 (3.9) |  |  |
| Site of TB |  |  |  | 0.256 (0.027–2.393) | 0.202 |  |  | 0.829 (0.087–7.869) | 0.870 |  |  | 2.000 (0.201–19.914) | 0.547 |
| Pulmonary | 138 (95.8) | 40 (97.6) | 41 (91.1) |  |  | 19 (95.0) | 63 (94.0) |  |  | 9 (90.0) | 72 (94.7) |  |  |
| Extrapulmonary | 6 (4.2) | 1 (2.4) | 4 (8.9) |  |  | 1 (5.0) | 4 (6.0) |  |  | 1 (10.0) | 4 (5.3) |  |  |
| New treatment | 104 (72.2) | 29 (70.7) | 33 (73.3) |  |  | 11(55.0) | 51 (85.1) |  |  | 6 (60.0) | 56 (73.7) |  |  |
| Retreatment | 40 (27.8) | 12 (29.3) | 12 (26.6) |  |  | 9 (45.0) | 16 (23.9) |  |  | 4 (40.0) | 20 (26.3) |  |  |
| HIV infection | 15 (10.4) | 4 (9.8) | 7 (15.6) | 1.704 (0.460–6.310) | 0.421 | 1 (5.0) | 10 (14.9) | 3.333 (0.400–27.776) | 0.241 | 1 (10.0) | 10 (13.2) | 1.364 (0.156–11.948) | 0.779 |
| Previous TB | 40 (27.8) | 11 (26.8) | 11 (24.4) | 0.882 (0.335–2.326) | 0.800 | 9 (45.0) | 14 (20.9) | 3.097 (1.073–8.937) | **0.032** | 5 (50.0) | 17 (22.4) | 0.288 (0.075–1.114) | 0.060 |
| Previous TB exposure | 46 (31.9) | 12 (29.3) | 13 (28.9) | 0.982 (0.387–2.493) | 0.969 | 5 (25.0) | 20 (29.9) | 1.277 (0.409–3.989) | 0.674 | 1 (10.0) | 23 (30.3) | 3.906 (0.467–32.641) | 0.179 |
| Presenting symptoms | | | | | | | | | | | | | |
| Poor general condition | 123 (85.4) | 34 (82.9) | 36 (80.0) | 0.824 (0.276–2.457) | 0.728 | 16 (80.0) | 55 (82.1) | 1.146 (0.325–4.044) | 0.832 | 9 (90.0) | 61 (80.3) | 0.452 (0.053–3.847) | 0.457 |
| Cough | 120 (83.3) | 34 (82.9) | 38 (84.4) | 1.118 (0.356–3.513) | 0.849 | 16 (35.5) | 56 (83.6) | 1.273 (0.357–4.542) | 0.710 | 8 (80.0) | 64 (84.2) | 1.333 (0.252–7.066) | 0.735 |
| Weight loss | 115 (79.9) | 33 (80.5) | 37 (82.2) | 1.121 (0.378–3.323) | 0.836 | 17 (37.7) | 54 (80.6) | 0.733 (0.187–2.881) | 0.656 | 9 (90.0) | 62 (81.6) | 0.492 (0.058–4.207) | 0.509 |
| Fever | 113 (78.5) | 33 (80.5) | 37 (82.2) | 1.121 (0.378–3.323) | 0.836 | 15 (33.3) | 55 (82.1) | 1.528 (0.465–5.018) | 0.483 | 7 (70.0) | 62 (81.6) | 1.898 (0.436–8.268) | 0.387 |
| Expectoration | 110 (76.4) | 30 (73.2) | 32 (71.1) | 0.903 (0.351–2.322) | 0.832 | 15 (33.3) | 47 (70.1) | 0.783 (0.251–2.448) | 0.674 | 8 (80.0) | 54 (71.1) | 0.614 (0.121–3.122) | 0.553 |
| Chills | 105 (72.9) | 31 (75.6) | 33 (73.3) | 0.887 (0.336–2.345) | 0.809 | 15 (33.3) | 50 (74.6) | 0.980 (0.310–3.103) | 0.973 | 6 (6.0) | 58 (76.3) | 2.148 (0.545–8.464) | 0.266 |
| Diaphoresis | 95 (66.0) | 28 (68.3) | 25 (55.6) | 0.580 (0.240–1.402) | 0.225 | 13 (28.8) | 40 (59.7) | 0.798 (0.282–2.258) | 0.670 | 6 (60.0) | 46 (60.5) | 1.022 (0.266–3.928) | 0.974 |
| Dyspnea | 92 (63.9) | 25 (61.0) | 26 (57.8) | 0.876 (0.37–2.075) | 0.763 | 13 (28.8) | 38 (56.7) | 0.706 (0.250–1.993) | 0.509 | 7 (70.0) | 43 (56.6) | 0.558 (0.134–2.325) | 0.419 |
| Hemoptysis | 40 (27.8) | 8 (19.5) | 9 (20.0) | 1.031 (0.356–2.986) | 0.955 | 5 (25.0) | 12 (17.9) | 0.655 (0.199–2.150) | 0.483 | 3 (30.0) | 14 (18.4) | 0.527 (0.121–2.295) | 0.387 |
| Chest pain | 32 (22.2) | 8 (19.5) | 14 (31.1) | 1.863 (0.687–5.051) | 0.218 | 5 (25.0) | 17 (25.4) | 1.020 (0.322–3.228) | 0.973 | 3 (30.0) | 19 (25.0) | 0.778 (0.183–3.312) | 0.733 |
| Diarrhea | 30 (20.8) | 6 (14.6) | 13 (28.9) | 2.370 (0.805–6.976) | 0.112 | 2 (10.0) | 17 (25.4) | 3.060 (0.642–14.578) | 0.144 | 1 (10.0) | 18 (23.7) | 2.793 (0.331–23.565) | 0.327 |
| Dysphonia | 20 (13.9) | 5 (12.2) | 6 (13.3) | 1.108 (0.311–3.946) | 0.875 | 3 (6.7) | 8 (11.9) | 0.768 (0.183–3.218) | 0.718 | 1 (10.0) | 10 (13.2) | 1.364 (0.156–11.948) | 0.779 |
| Abdominal pain | 18 (12.5) | 6 (14.6) | 7 (15.6) | 1.075 (0.329–3.508) | 0.905 | 2 (10.0) | 11 (16.4) | 1.768 (0.358–8.734) | 0.480 | 2 (20.0) | 11 (14.5) | 0.677 (0.127–3.618) | 0.647 |
| Dysphagia | 16 (11.1) | 4 (9.8) | 5 (11.1) | 1.156 (0.288–4.636) | 0.838 | 3 (6.7) | 6 (9.0) | 0.557 (0.126–2.464) | 0.436 | 1 (10.0) | 8 (10.5) | 1.059 (0.118–9.480) | 0.959 |
| Lymphadenopathy | 14 (9.7) | 6 (14.6) | 5 (11.1) | 0.729 (0.205–2.598) | 0.625 | 3 (6.7) | 8 (11.9) | 0.768 (0.218–3.218) | 0.718 | 2 (20.0) | 9 (11.8) | 0.537 (0.098–2.937) | 0.468 |
| Altered levels of consciousness | 12 (8.3) | 2 (4.9) | 2 (4.4) | 0.907 (0.122–6.751) | 0.924 | 1 (5.0) | 3 (4.5) | 0.891 (0.087–9.066) | 0.922 | 1 (10.0) | 3 (3.9) | 0.370 (0.035–3.944) | 0.393 |
| Back pain | 11 (7.6) | 2 (4.9) | 5 (11.1) | 2.438 (0.446– 13.317) | 0.291 | 2 (10.0) | 5 (7.5) | 0.726 (0.130–4.060) | 0.714 | 1 (10.0) | 6 (7.9) | 0.771 (0.083–7.159) | 0.819 |
| Headache | 9 (6.3) | 2 (4.9) | 5 (11.1) | 2.438 (0.446–13.317) | 0.291 | 0 (0.0) | 7 (10.4) | 1.333 (1.175–1.513) | 0.132 | 0 (0.0) | 7 (9.2) | 1.145 (0.053–1.245) | 0.317 |
| Cachexia | 5 (3.5) | 1 (2.4) | 3 (6.7) | 2.857 (0.285–28.618) | 0.352 | 0 (0.0) | 4 (6.0) | 1.317 (1.167–1.487) | 0.263 | 0 (0.0) | 4 (5.3) | 1.139 (1.051–1.235) | 0.458 |
| Seizures | 3 (2.1) | 0 (0.0) | 2 (4.4) | 1.953 (1.585–2.407) | 0.172 | 0 (0.0) | 2 (3.0) | 1.308 (1.162–1.471) | 0.434 | 0 (0.0) | 2 (2.6) | 1.135 (1.049–1.228) | 0.604 |
| Co-morbidities | | | | | | | | | | | | | |
| Smoking | 91 (63.2) | 28 (68.3) | 28 (62.2) | 0.765 (0.313–1.866) | 0.555 | 11 (55.0) | 45 (67.2) | 1.674 (0.605–4.631) | 0.319 | 6 (60.0) | 50 (67.1) | 1.282 (0.332–4.950) | 0.718 |
| Alcohol use | 77 (53.5) | 22 (53.7) | 26 (57.7) | 1.182 (0.504–2.772) | 0.701 | 12 (60.0) | 36 (53.7) | 0.774 (0.280–2.137) | 0.621 | 7 (70.0) | 41 (53.9) | 0.502 (0.121–2.089) | 0.337 |
| Drug use | 57 (39.6) | 15 (36.6) | 16 (35.6) | 0.956 (0.396–2.308) | 0.921 | 7 (35.0) | 24 (22.5) | 1.307 (0.364–2.950) | 0.946 | 3 (30.0) | 28 (36.8) | 1.361 (0.326–5.691) | 0.672 |
| DM | 38 (26.4) | 13 (31.7) | 13 (28.9) | 0.875 (0.348–2.198) | 0.776 | 6 (30.0) | 21 (31.3) | 1.065 (0.359–3.158) | 0.909 | 3 (30.0) | 23 (30.3) | 1.013 (0.240–4.267) | 0.986 |
| Incarceration | 24 (16.7) | 6 (14.6) | 5 (11.1) | 0.729 (0.205–2.598) | 0.625 | 1 (5.0) | 10 (14.92) | 3.333 (0.400–27.776) | 0.241 | 0 (0.0) | 11 (14.5) | 1.154 (1.056–1.261) | 0.198 |
| Radiological characteristics | | | | | | | | | | | | | |
| Chest radiography pattern | 132 (91.7) | 35 (85.4) | 42 (93.3) | 2.400 (0.559–10.300) | 0.228 | 15 (75.0) | 63 (94.0) | 0.190 (0.037–0.955) | **0.014** | 7 (70.0) | 71 (93.4) | 6.086 (1.194–31.011) | **0.017** |
| Nodule | 77 (53.5) | 17 (41.5) | 27 (60.0) | 2.118 (0.895–5.011) | 0.086 | 13 (65.0) | 29 (43.3) |  | 0.064 | 3 (30.0) | 42 (55.3) | 2.882 (0.692–11.997) | 0.133 |
| Cavitary | 63 (43.8) | 21 (51.2) | 12 (26.7) | 2.888 (1.173–7.108) | **0.019** | 7 (35.0) | 26 (38.8) | 1.178 (0.415–3.339) | 0.758 | 4 (40.0) | 29 (38.2) | 0.926 (0.241–3.560) | 0.910 |
| Interstitial | 54 (37.5) | 16 (39.0) | 13 (28.9) | 0.635 (0.258–1.561) | 0.321 | 5 (25.0) | 24 (35.8) | 1.674 (0.542–5.176) | 0.368 | 3 (30.0) | 26 (34.2) | 1.213 (0.289–5.086) | 0.791 |
| Consolidation | 41 (28.5) | 10 (24.4) | 13 (28.9) | 1.259 (0.482–3.293) | 0.638 | 4 (20.0) | 20 (29.9) | 1.702 (0.505–5.732) | 0.387 | 1 (10.0) | 23 (30.3) | 3.906 (0.467–32.641) | 0.179 |
| Pleural effusion | 15 (10.4) | 5 (12.2) | 5 (11.1) | 0.900 (0.241–3.365) | 0.876 | 3 (15.0) | 7 (10.4) | 0.661 (0.154–2.835) | 0.575 | 2 (20.0) | 8 (10.5) | 0.471 (0.085–2.612) | 0.380 |
| Miliary | 14 (9.7) | 5 (12.2) | 3 (6.7) | 0.514 (0.115–2.303) | 0.378 | 2 (10.0) | 6 (9.0) | 0.885 (0.164–4.771) | 0.887 | 1 (10.0) | 7 (9.2) | 0.913 (0.100–8.302) | 0.936 |
| CT characteristics | 129 (89.6) | 36 (87.8) | 38 (84.4) | 0.754 (0.219–2.592) | 0.653 | 16 (80.0) | 59 (88.1) | 1.844 (0.492–6.911) | 0.359 | 8 (80.0) | 66 (86.8) | 1.650 (0.306–8.908) | 0.557 |
| Nodules | 99 (68.8) | 27 (65.9) | 31 (68.9) | 1.148 (0.466–2.832) | 0.764 |  |  |  |  | 6 (60.0) | 52 (68.4) | 1.444 (0.373–5.597) | 0.593 |
| Apical asymmetry | 95 (66.0) | 28 (68.3) | 27 (60.0) | 0.696 (0.287–1.692) | 0.424 | 11 (55.0) | 44 (65.7) | 1.565 (0.567–4.319) | 0.385 | 6 (60.0) | 48 (63.2) | 1.143 (0.297–4.401) | 0.846 |
| Cavitary | 84 (58.3) | 24 (58.5) | 16 (35.6) | 2.559 (1.071–6.114) | **0.033** | 9 (45.0) | 31 (46.3) | 1.052 (0.386–2.860) | 0.920 | 5 (50.0) | 34 (44.7) | 0.810 (0.216–3.029) | 0.753 |
| Miliary | 16 (11.1) | 6 (14.6) | 6 (13.3) | 0.897 (0.265–3.040) | 0.862 | 2 (10.0) | 10 (14.9) | 1.579 (0.316–7.883) | 0.575 | 1 (10.0) | 11 (14.5) | 1.523 (0.175–13.240) | 0.701 |

CI: confidence intervals; CT: computed tomography; DR: drug resistance; DM: diabetes mellitus; EMB: ethambutol; INH: isoniazid; MDR: multidrug resistance; PZA: pyrazinamide; R: resistant; RIF: rifampicin; S: susceptible; SD: standard deviation, STR: streptomycin; OR: odds ratio; TB: *M. tuberculosis*. Numbers in bold indicate a statistically significant p-value (*p* < 0.05). 3
